# Extracellular Vesicles of Human Periodontal Ligament Stem Cells Contain MicroRNAs Associated to Proto-Oncogenes: Implications in Cytokinesis

**DOI:** 10.3389/fgene.2020.00582

**Published:** 2020-06-04

**Authors:** Luigi Chiricosta, Serena Silvestro, Agnese Gugliandolo, Guya Diletta Marconi, Jacopo Pizzicannella, Placido Bramanti, Oriana Trubiani, Emanuela Mazzon

**Affiliations:** ^1^IRCCS Centro Neurolesi “Bonino-Pulejo”, Messina, Italy; ^2^Department of Medical, Oral and Biotechnological Sciences, University “G. d’Annunzio” Chieti and Pescara, Chieti, Italy; ^3^ASL02 Lanciano-Vasto-Chieti, “Ss. Annunziata” Hospital, Chieti, Italy

**Keywords:** human periodontal ligament stem cells, extracellular vesicles, next generation sequencing, non-coding RNAs, microRNAs, cytokinesis

## Abstract

The human Periodontal Ligament Stem Cells (hPDLSCs) exhibit self-renewal capacity and clonogenicity potential. The Extracellular Vesicles (EVs) secreted by hPDLSCs are particles containing lipids, proteins, mRNAs, and non-coding RNAs, among which microRNAs, that are important in intercellular communication. The purpose of this study was the analysis of the non-coding RNAs contained in the EVs derived from hPDLSCs using Next Generation Sequencing. Moreover, our data were enriched using bioinformatic tools. The analysis highlighted the presence of non-coding RNAs and five microRNAs: *MIR24-2*, *MIR142*, *MIR335*, *MIR490*, and *MIR296*. Our results show that these miRNAs target the genes classified in two terms of the Gene Ontology: “Ras protein signal transduction” and “Actin/microtubule cytoskeleton organization.” Noteworthy, the in-deep analysis of our EVs highlights that the miRNAs could be implicated in the silencing of proto-oncogenes involved in 12 different types of tumors.

## Introduction

The human Periodontal Ligament Stem Cells (hPDLSCs) are mesenchymal stem cells that can be easily harvested from periodontal tissue. Non-invasive surgery during standard dental scaling does not entail any additional risk to the donor (Chiricosta et al., 2019). The hPDLSCs showed self-renewal capacity, differentiation and immunomodulatory proprieties (Eleuterio et al., 2013). The Extracellular Vesicles (EVs) are particles delimited by a lipid bilayer capable of crossing biological barriers and being internalized in the target cells so that they play an essential role in intercellular communication (Valadi et al., 2007; Pizzicannella et al., 2019b). EVs contain different macromolecules such as lipids, proteins, DNA, mRNA and non-coding RNAs (ncRNAs) among which microRNAs (or miRNAs) (Zhang et al., 2019). The dynamic light-scattering analysis, previously demonstrated from our research group, highlighted the presence of two different populations of EVs derived from hPDLSCs. The average diameter of the EVs populations is between 90 ± 20 nm and 1,200 ± 400 nm and they have the same ζ-potential of −10.7 ± 0.9 mV. The tapping-mode topographic 3D atomic force-microscopy measurements showed that EVs derived from hPDLSCs have a globular shape with a central depression and a relatively smooth surface (Diomede et al., 2018).

The ncRNAs are circulating RNAs that represent the 99% of total RNAs and can be classified according with length (small 18–200 nucleotides and long >200), with function (ribosomal RNAs and transfer RNAs), or with regulation [miRNAs and long non-coding RNAs (lncRNAs)] (Dozmorov et al., 2013; Palazzo and Lee, 2015). The ncRNAs are involved in gene regulation by RNA interference, RNA modification or spliceosomal cycle. Specifically, miRNAs are small ncRNAs (18–25 nucleotides in length) that prevent the translation of mRNAs and consequently contribute to define the amount of proteins inside the cell (Bartel, 2004). miRNAs play an important role in physiological functions such as cell-cell communication, cell proliferation and vascularization. The ncRNAs and miRNAs are implicated in various diseases including cancer, in which they play an important biological role (Slaby et al., 2017).

Therefore, in this study, using Next Generation Sequencing (NGS), we want to analyze the ncRNAs and miRNAs contained in EVs derived from hPDLSCs in the early stages of stemness. Particularly, we investigated the possible implications of ncRNAs in the biological processes and in the proto-oncogenes modulation.

## Materials and Methods

### Culture and Extraction of the hPDLSCs

All the subjects that were involved in the study gave their informed consent. The protocol executed for this study was approved by the Medical Ethics Committee at the Medical School, “G. d’Annunzio” University of Chieti-Pescara, Chieti, Italy (n°266 17 April 2014) and it is in accordance with the Declaration of Helsinki. Five patients in healthy general conditions were chosen for tooth removal for orthodontic purposes. Subsequently, the cells were cultured in the Mesenchymal Stem Cell Growth Medium Chemically Defined (MSCGM-CD) Bulletin medium (Lonza, Basel, Switzerland). In order to minimize exposure to the non-human substances and facilitate the growth of human MSCs, the MSCGM-CD was changed twice a week. Cells isolation and characterization were performed as previously described (Rajan et al., 2016; Trubiani et al., 2016). The hPDLSCs were collected and washed with PBS (Lonza) several times. Finally, they were cultured at 37°C with 5% of CO_2_ with the MSCGM-CD medium.

### Isolation of the hPDLSCs-Derived EVs

The hPDLSCs, isolated from each patient, were collected from conditioned medium (CM; 10 mL) at passage 2 after 48 h of incubation. For 15 min, the CM was centrifuged at 3,000 × g in order to remove debris and suspended cells. EVs were extracted using the commercial agglutinant ExoQuick TC (System Biosciences, Euroclone SpA, Milan, Italy). Specifically, 10 mL of CM of the hPDLSCs were mixed with 2 mL ExoQuick TC and the whole was incubated at 4°C overnight without rotation. Subsequently, in order to deposit the EVs, a centrifugation step was carried out for 30 min at 1,500 × g. Finally, the pellets were resuspended in 200 μL of PBS (Ca^2+^ and Mg^2+^). The EVs, obtained from hPDLSCs of each patient, were used for transcriptomic analyses.

### RNA Extraction and Non-coding Analysis

The RNA extraction and processing were conducted as previously reported (Silvestro et al., 2020). Briefly, the Total Exosome RNA and Protein Isolation Kit (catalog #4478545; Thermo Fisher Scientific, Rockford, IL, United States) was used to isolate the RNA following the manufacturer’s protocol and 30 μL of RNA solution were collected from each sample (Pizzicannella et al., 2019a).

The manufacturer instructions and the TruSeq RNA Exome protocol (Illumina, San Diego, CA, United States) were followed in order to prepare the library. The normalized libraries were analyzed using the MiSeq instrument (Illumina) in a single read mode. For the NGS analysis, the RNA extracted from the EVs of each patient was repeated in triplicate.

The software fastQC (Babraham Institute, Cambridge, United Kingdom) was used to perform the quality check of the reads. Then, the adapters and the low-quality bases were identified and removed by the software Trimmomatic (Usadel Lab, Aachen, Germany) (Bolger et al., 2014). The “Homo Sapiens” reference genome, available from the University of California Santa Cruz website^1^, was used to align the reads taking advantage of the software Spliced Transcripts Alignment to a Reference RNA-seq aligner (STAR) (Dobin et al., 2013). Finally, the Cufflinks software (Trapnell Lab, Washington, DC, United States), version 2.2.1, was used to assign the gene symbol to each transcript (Trapnell et al., 2013). In order to study the transcripts, the whole set was classified with HUGO Gene Nomenclature Committee website^2^ (Stelzer et al., 2016) using the “non-coding RNAs” group (475). Specifically, the genes that are targeted by the identified miRNAs were analyzed with TargetScanHuman (version 7.2) (Agarwal et al., 2015). Moreover, the target genes were enriched with the “Biological Process” terms included in the “Gene Ontology” using PANTHER. Finally, the Human microRNA Disease Database (HMDD) v3.2 was used in order to associate the genes target of our miRNAs to the disease in which their expression is deregulated. All the plots were depicted with the software R.

## Results

### Evaluation of Non-coding Transcripts of EVs Derived From hPDLSCs

**FIGURE 1:**
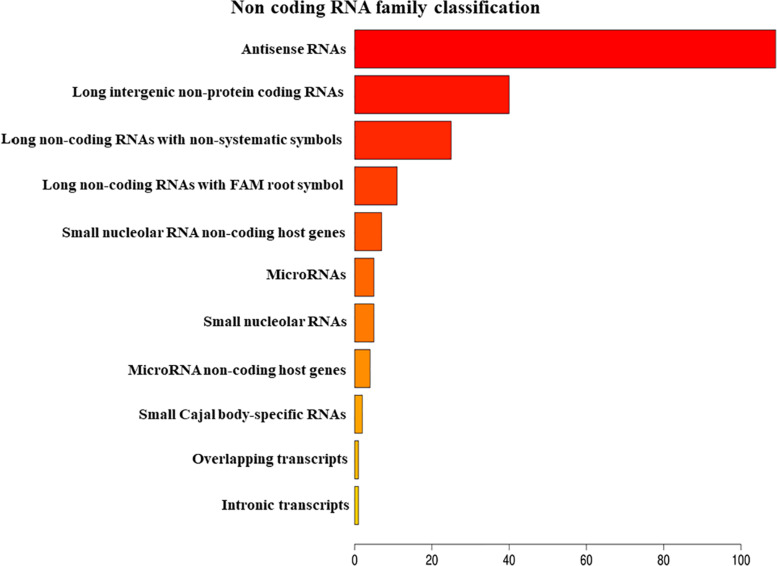
Classification of the non-coding RNAs families contained in EVs belong using HUGO database.

**FIGURE 2:**
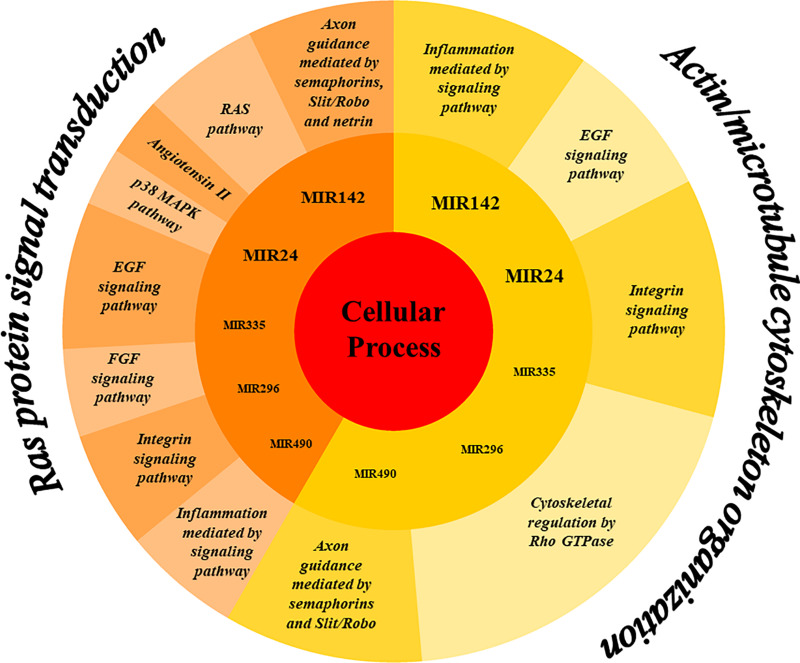
Representation of the biological processes in which the genes targeted by the miRNAs in EVs are mainly involved. The miRNAs *MIR24*, *MIR142*, *MIR335*, *MIR296*, and *MIR490* mainly target genes belonging to the “Cellular processes” category of the Gene Ontology. More specifically, the genes fall into two sub-categories “Ras protein signal transduction” and “Actin/microtubule cytoskeleton organization.” The genes involved in these subprocesses are characteristic of the pathways represented in the outer ring.

**FIGURE 3:**
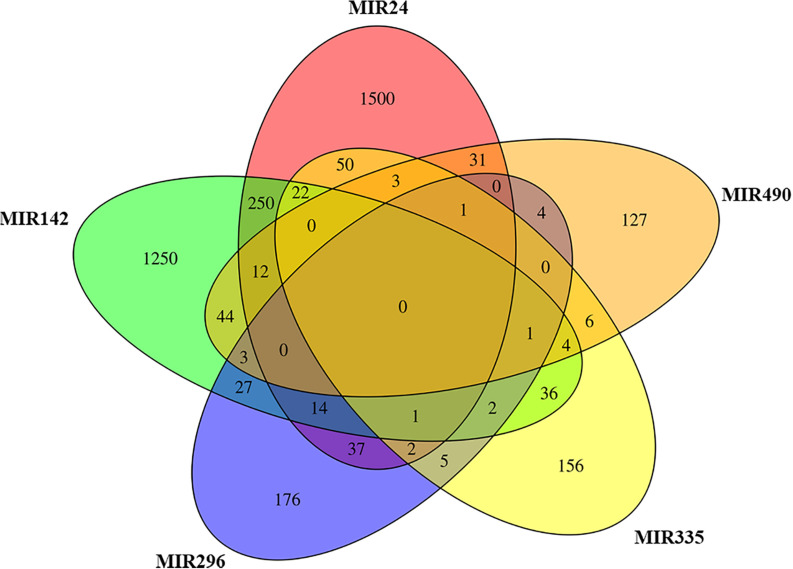
Venn diagram. The plot represents for each miRNA in EVs the genes that inhibits in comparison to the others. *MIR24* and *MIR142* target exclusively the highest number of genes.

The analysis of EVs derived from hPDLSCs reveals 955 non-coding transcripts (Supplementary Table S1). The HUGO website characterizes 212 transcripts distributed in 11 groups as showed in Figure 1. The most of the non-coding transcripts of EVs belong to the “Antisense RNAs” (109), to the “Long intergenic non-protein coding RNAs” (40) and to the “Long non-coding RNAs with non-systematic symbols” (25). The remaining transcripts are included in smaller categories. Our EVs contain also 5 miRNAs: *MIR24-2*, *MIR142*, *MIR296*, *MIR335*, *MIR490*. We studied the biological roles of our miRNAs using TargetScanHuman (Agarwal et al., 2015) that associates each miRNA to its target genes. We used the database PANTHER (Thomas et al., 2003) to visualize all the “Biological Process” terms in which the target genes are involved (Figure 2). The deepest terms that better characterize our transcripts are related to the cytoskeletal organization (“Actin/microtubule cytoskeleton organization”) and the signal transduction mediated by Ras protein (“Ras protein signal transduction”). PANTHER was also used to associate the genes with the pathway in which they are involved. In particular, the most characterized pathways include the genes *CDC42*, *RAC1*, *RHOC*, *RHOA*, *RHOJ*, *SPRY3*, *PFN2*, *WASL*, *TUBB*, *TUBB4B*, *SSH3*, *SSH1*, *ARPC5L*, *PARVA*, *RHOQ.* The first five genes are in common to “Actin/microtubule cytoskeleton organization” and “Ras protein signal transduction.” *SPRY3* is found only in the pathway related to Ras protein while the other genes are exclusively related to the cytoskeletal organization. Moreover, we inspected the amount of genes that are simultaneously targeted by our miRNAs as represented in the Venn Diagram in Figure 3. There is no gene that is targeted by all the miRNAs. Nevertheless, we observed that one gene (*GABRB1*) is not targeted just by *MIR142*, one gene (*ONECUT2*) just by *MIR24*, one gene (*CNNM2*) just by *MIR490*. Furthermore, *MIR-24* targets the most of the genes (1,500) immediately followed by *MIR142* (1,250). *MIR490*, *MIR335*, and *MIR296* target exclusively less than 200 genes each. Furthermore, using HMDD (Huang et al., 2019) we reported in Table 1 the associations found among our miRNAs and the target genes deregulated in specific diseases.

**TABLE 1:** MiRNAs found in hPDLSCs-derived EVs associated to specific cancer.

MiRNA	Target gene	Gene name	Disease	References
MIR24-2	S100A8	S100 calcium binding protein A8	Laryngeal carcinoma	Guo et al., 2012
MIR24-2	AGPAT2	1-acylglycerol-3-phosphate O-acyltransferase 2	Osteosarcoma	Song et al., 2013
MIR142	RAC1	Rac family small GTPase 1	Hepatocellular carcinoma	Wu et al., 2011
MIR142	PROM1	Prominin 1	Colon cancer	Shen et al., 2013
MIR142	ABCG2	ATP binding cassette subfamily G member 2	Colon cancer	Shen et al., 2013
MIR142	LGR5	Leucine rich repeat containing G protein-coupled receptor 5	Colon cancer	Shen et al., 2013
MIR142	HMGA1	High mobility group AT-hook 1	Osteosarcoma	Xu et al., 2014
MIR296	HMGA1	High mobility group AT-hook 1	Prostate cancer	Wei et al., 2011
MIR296	PIN1	Peptidylprolyl cis/trans isomerase, NIMA-interacting 1	Prostate cancer	Lee et al., 2014
MIR296	FGFR1	Fibroblast growth factor receptor 1	Hepatocellular carcinoma	Wang et al., 2016
MIR296	AKT2	AKT serine/threonine kinase 2	Pancreatic cancer	Li et al., 2017
MIR296	PLK1	Polo like kinase 1	Lung cancer	Xu et al., 2016
MIR296	SP1	Sp1 transcription factor	Cervical cancer	Lv and Wang, 2018
MIR296	CDX1	Caudal type homeobox 1	Gastric cancer	Li et al., 2014
MIR490	CCND1	Cyclin D1	Lung cancer	Gu et al., 2014
MIR490	CDK1	Cyclin dependent kinase 1	Ovarian cancer	Chen et al., 2015
MIR490	FOS	Fos proto-oncogene, AP-1 transcription factor subunit	Bladder cancer	Li et al., 2013
MIR490	HMGA2	High mobility group AT-hook 2	Osteosarcoma	Liu et al., 2015
MIR335	BCL2L2	BCL2 like 2	Gastric cancer	Xu et al., 2012
			Ovarian cancer	Cao et al., 2013
MIR335	SP1	Sp1 transcription factor	Gastric cancer	Xu et al., 2012
MIR335	ROCK1	Rho associated coiled-coil containing protein kinase 1	Osteosarcoma	Wang et al., 2013
			Neuroblastoma	Lynch et al., 2012
MIR335	MAPK1	Mitogen-activated protein kinase 1	Neuroblastoma	Lynch et al., 2012
MIR335	LRG1	Leucine rich alpha-2-glycoprotein 1	Neuroblastoma	Lynch et al., 2012

**To each miRNA found in hPDLSC-derived EVs is associated a Target Gene and the kind of cancer in which it is found deregulated inside the Human MicroRNA Disease Database along with the Reference in which it is described.**

## Discussion

EVs are phospholipids membrane-enclosed organizations that carry proteins, lipids and nucleic acids including mRNAs and ncRNAs (Trubiani et al., 2019).

In this study, we analyzed the ncRNAs contained in EVs derived from hPDLSCs at early stage of stemness with a focus on miRNAs.

Our results show that the ncRNAs contained in EVs are classified in 11 families (Figure 1). The most represented class of ncRNAs is the “Antisense RNAs,” small molecules that regulate gene’s expression by binding the complementary mRNAs (Xu et al., 2018). Our results show also the presence of lncRNAs, an important class of transcripts that indirectly regulates the transcription recruiting transcription factors and affecting mRNA stability (Fatima and Nawaz, 2017). EVs transport lncRNAs from one cell to another and can induce epigenetic modifications in the receiving cells (Fatima and Nawaz, 2017). Other classes of ncRNAs represented in our EVs are the small nucleolar RNAs and miRNAs. The small nucleolar RNAs are involved in the chemical alteration of different RNAs and in the regulation of the alternative splicing in pre-mRNAs (Decatur and Fournier, 2002; Falaleeva et al., 2016; Sharma et al., 2016).

Among ncRNAs, miRNAs are the most studied since they can regulate the expression of 60% of the human genes. Specifically, miRNAs are post-transcriptional regulators that bind complementary mRNA and consequently reduce protein expression. miRNAs, released from the EVs, can determine the cell fate through their participation in differentiation and in the regulation of reprogramming processes (Friedman et al., 2009; Fatima and Nawaz, 2017; Mens and Ghanbari, 2018). In the last two decades, many studies contextualized the role of miRNAs in cancer development (Rupaimoole and Slack, 2017). Interestingly, in our EVs, we found five miRNAs: *MIR24-2*, *MIR142*, *MIR335*, *MIR490*, and *MIR296*.

Our bioinformatic analysis, represented in Figure 3, highlights the amount of target genes that are modulated by our miRNAs. Indeed, both *MIR24-2* and *MIR142* are able to influence more than 1,000 genes while *MIR335*, *MIR490*, and *MIR296* less than 200 each. The representation with the Venn Diagram shows that each miRNA can regulate several genes and each gene can be regulated by more than one miRNA. This evidence suggests that *MIR24-2* and *MIR142* are ones most involved.

The *MIR24-2*, *MIR142*, *MIR335*, *MIR490*, and *MIR296* found in our EVs target the genes mainly involved in “Ras protein signal transduction” and “Actin/microtubule cytoskeleton organization” (Figure 2).

The Ras proteins are GTPases that act as switches recruiting either the Map Kinases or the RHO/RAC family resulting in downstream signaling for transcription, cell cycle or cytoskeletal organization (Bustelo et al., 2007).

The analysis performed using PANTHER database shows that our miRNAs target *CDC42*, *RAC1*, *RHOA*, *RHOC*, and *RHOJ* that simultaneously participate in the regulation of “Actin/microtubule cytoskeleton organization” and “Ras protein signal transduction.” These genes encode for smalls GTPases/Rho family that are active when bound to Guanosine Triphosphate (GTP) or inactive when bound to Guanosine Diphosphate (GDP). The *CDC42*, *RAC1*, *RHOC*, and *RHOA* genes play an important role in cytoskeletal rearrangement, morphology regulation, cell motility, cell adhesion and cell cycle regulation (Aspenström, 2019; Thomas et al., 2019). *RHOJ* is a gene that encodes for an endothelial member of the Cdc42 subfamily involved in endothelial motility, tubulogenesis and microtubule lumen formation (Leszczynska et al., 2011). Indeed, miRNAs are very important regulators of these GTPase Rho family (Liu et al., 2012).

Additionally, as regards the Gene Ontology term “Ras protein signal transduction,” the miRNAs inside our EVs target the *SPRY3* gene, which encodes for the Protein sprouty homolog 3 (Spry3), an intracellular negative regulators of Receptor tyrosine kinase signaling and of the Fibroblast Growth Factor (FGF) pathways (Hacohen et al., 1998). Furthermore, Panagiotaki et al. (2010) showed that Spry3 negatively regulates the release of calcium downstream of brain-derived neurotrophic factor signaling, and consequently cause the inhibition of the axons branching in cultured cortical neurons.

Moreover, the miRNAs present in our EVs regulate the “Actin/microtubule cytoskeleton organization” process by targeting different genes such as *TUBB*, *TUBB4B*, *PFN2*, *WASL*, *SSH3*, *SSH1*, *ARPC5L*, *PARVA*, and *RHOQ*. In detail, *TUBB* and *TUBB4B* encode respectively for a beta-tubulin protein and for the beta-4B tubulin chain, both representing structural components of the microtubules. *RHOQ* encodes for a small GTPase involved in the regulation of the cell shape through the organization of the actin cytoskeleton. *SSH1* and *SSH3* encode for protein phosphatases that play a role in the regulation of the dynamics of the actin filament. In addition, *SSH1* and *SSH3* activate the actin-binding protein family ADF/cofilin that promotes the disassembling of the actin filaments (Kurita et al., 2007). *ARPC5L* gene, part of Arp2/3 complex, encodes for the Actin Related Protein 2/3 Complex Subunit 5 Like. This gene regulates the actin polymerization in the cytoskeleton and generates an actin filament network (Abella et al., 2016). *PARVA* encodes for the α-parvin protein that is involved in the regulation of the actin cytoskeleton dynamism (Sepulveda and Wu, 2006). *PFN2* is an actin-binding protein that regulates the architecture of the synapse and the cytoskeleton (Jeong et al., 2018). *WASL*, which encodes for Neural Wiskott-Aldrich syndrome protein, appears to be involved in the polymerization of actin. In addition, *PFN2* and *WASL* are highly expressed in neural tissues and participate to the axon guidance in the Slit/Robo pathway. These results show that our EVs contain miRNAs involved in axon guidance and consequently in neural differentiation.

Moreover, we evaluated (using HMDD) how much our miRNAs deregulate oncogenes involved in different kinds of cancers. Noteworthy, our miRNAs are involved at least in 12 different cancers (Table 1) and specifically in their development and progression (Calin and Croce, 2006).

The *MIR24-2* encodes miR-24 that plays an important role as a suppressor of the genes *E2F2* and *MYC.* Moreover, by binding the complementary 3′-UTR mRNA of these genes, miR24-2 is involved in the regulation of the cell cycle (Lal et al., 2009). Guo et al. (2012) also showed that the up-regulation of *MIR24* leads to morphological changes, low cell proliferation and enhancement of cell invasion potential in laryngeal squamous cell carcinoma by inhibiting transcription of the S100A8 gene. Additionally, the overexpression of *MIR24* inhibits osteosarcoma cell proliferation by blocking the transcription of *AGPAT2* gene encoding for Lysophosphatidic Acid Acyltransferase β, an enzyme involved in osteosarcoma cell proliferation (Song et al., 2013).

The *MIR142* appears to be a potential proto-oncogene suppressor. Indeed, through the transcriptional block of *RAC1*, a gene that encodes for a GTPase that regulates cell growth and migration and promotes the activation of protein kinases. Wu et al. (2011) showed that *MIR142* has also a role in suppressing migration and invasion of hepatocellular carcinoma cells. In addition, *MIR142* inhibits the proliferation of pancreatic cancer cells by decreasing the expression of heat shock protein 70 (Mackenzie et al., 2013). It inhibits the growth of cells responsible for colon cancer, hindering the transcription of *PROM1* (CD133), *ABCG2*, and *LRG5* genes (Shen et al., 2013). Moreover, by blocking the transcription of the *HMGA1* gene, an important gene involved in promoting cancer and increasing invasiveness, Xu et al. (2014) reveals that *MIR142* can act as a suppressor in osteosarcoma. Furthermore, as reported by Dickman et al. (2017) the *MIR142* is secreted by oral squamous cell carcinoma cells, promoting the growth of the tumor. The release of miR-142-3p also influences the tumor microenvironment, promoting angiogenesis (Dickman et al., 2017). Indeed, in oral squamous cell carcinoma, *MIR142* was up-regulated, demonstrating how the differential expression of miRNAs could be useful in the diagnosis of oral cancer (Xu et al., 2019).

*HMGA1* gene can be also targeted by *MIR296*, another miRNA presents in our EVs, supporting the knowledge by which different miRNAs can interact with the same mRNA. Negative regulation of this gene by *MIR296* appears to reduce invasiveness in prostate cancer cells (Wei et al., 2011). In addition, *MIR296* targeting *PIN1*, another gene involved in tumor development, suppresses cell proliferation and growth in prostate cancer cells (Lee et al., 2014). In hepatocellular carcinoma, *MIR296* inhibits the proliferative capacity and progression of the cell cycle and induces apoptosis blocking the transcription of the *FGFR1* proto-oncogene (Wang et al., 2016). Other targets of *MIR296* are the proto-oncogenes *AKT2* in pancreatic cancer (Li et al., 2017), *PLK1* in non-small cell lung cancer (Xu et al., 2016), *SP1* in cervical cancer (Lv and Wang, 2018) and *CDX1* in gastric cancer (Li et al., 2014). Through the negative regulation of the expression of these genes, *MIR296* exerts its action as a tumor suppressor. Therefore, EVs derived from hPDLSCs, through the secretion of *MIR296*, could act as suppressors of the malignant progression by attenuating the transcription of oncogenic targets. The *MIR296*, as reported by Severino et al. (2015) was also detected in the in metastases of patients with oral squamous cell carcinoma.

In the transcriptome of our EVs, we found also *MIR490* that seems to have the role of tumor suppressor in lung cancer cells A549 and ovarian cancer by blocking the transcription of *CCND1* and *CDK1* respectively, two important genes involved in cell cycle progression (Gu et al., 2014; Chen et al., 2015). Additionally, as showed by Li et al. (2013), the *MIR490* seems to be a suppressor of bladder cancer cell proliferation by blocking the transcription of *FOS*. Moreover, *MIR490* inhibits the expression of *HMGA2* and its downregulation appears to affect the development potential of the osteosarcoma (Liu et al., 2015).

*MIR335*, present in our EVs, also appears to play the role of proto-oncogene suppressor in a wide variety of cancers. It is known that *MIR335* negatively regulates the metastasis in gastric cancer by targeting *BCL2L2* and *SP1* (Xu et al., 2012). Likewise targeting the same *BCL2L2* gene, it is capable of inhibiting invasiveness in ovarian cancer (Cao et al., 2013). In neuroblastoma cells, *MIR335* downregulates the *ROCK1* and *MAPK1* genes, involved in the non-canonical TGF-β pathways. This modulation implicates a reduced invasiveness of the neuroblastoma cells. In addition, *MIR335* inhibits the mRNA transcribed by *LRG1* gene reducing the migration of neuroblastoma cells (Lynch et al., 2012). Similarly, it negatively regulates at the post-transcriptional level the *ROCK1* inhibiting tumor cell invasion and migration in osteosarcoma (Wang et al., 2013). Conversely, Kabir et al. (2016) showed that *MIR335* is up-regulated in fibroblasts associated with senescent cancer. These cells present in the tumor environment of oral neoplasms. Furthermore, the authors reported that *MIR335* is also involved in the development of a secretory phenotype associated with senescence. In this way, *MIR335* contributing to the progression of cancer (Kabir et al., 2016).

Interestingly, in our previous manuscript, we have already demonstrated in an analysis of the oral stem cell transcriptome that, following the *in vitro* expansion, many oncogenes were absent or poorly expressed (Gugliandolo et al., 2017).

## Conclusion

The EVs derived from hPDLSCs contain several ncRNAs among which the five miRNAs: *MIR24-2*, *MIR142*, *MIR296*, *MIR335*, and *MIR490*. Specifically, these miRNAs regulate genes that are involved in “Ras protein signal transduction” and “Actin/microtubule cytoskeleton organization” processes that regulate cell growth and differentiation during cytokinesis. Moreover, our results demonstrate that *MIR24* and *MIR142* are the most relevant miRNAs because they target more than 1,000 genes each. Furthermore, all the miRNAs detected in our EVs could have a potential role as proto-oncogenes suppressors. These findings indicated that the EVs obtained from hPDLSCs can be considered as anticancer therapeutic agents. This evidence supports the role of mesenchymal stem cell derivatives in the deregulatory functioning of human carcinogenesis in addition to their use in regenerative medicine.

## Data Availability Statement

We uploaded our data to the repository Sequence Read Archive (accession number PRJNA630492).

## Ethics Statement

The study involving human participants was approved by the Medical Ethics Committee at the Medical School, “G. d’Annunzio” University of Chieti-Pescara, Chieti, Italy (n°266 17 April 2014) and it is in accordance with the Declaration of Helsinki. All the subjects that were involved in the study gave their informed consent.

## Author Contributions

JP and GM performed the hPDLSCs culturing and the isolation of the EVs. AG performed the RNA extraction and library preparation. LC performed the computational and statistical analysis. LC and SS wrote the manuscript. PB, OT, and EM designed the study and revised the manuscript.

## Conflict of Interest

The authors declare that the research was conducted in the absence of any commercial or financial relationships that could be construed as a potential conflict of interest.

## Footnotes

1 http://labshare.cshl.edu/shares/gingeraslab/www-data/dobin/STAR/STARgenomes/Old/ENSEMBL/homo_sapiens/ENSEMBL.homo_sapiens.release-75/

2 https://www.genenames.org/

## Abbreviations

EVs: Extracellular Vesicles
FGF: Fibroblast Growth Factor
GDP: Guanosine Diphosphate
GTP: Guanosine Triphosphate
hPDLSCs: human Periodontal Ligament Stem Cells
lncRNAs: long non-coding RNAs
miRNAs: microRNAs
MSCGM-CD: Mesenchymal Stem Cell Growth Medium Chemically Defined
ncRNAs: non-coding RNAs
NGS: Next Generation Sequencing
Spry3: protein sprout homolog 3
STAR: Spliced Transcripts Alignment to a Reference RNA-seq aligner
HMDD: Human microRNA Disease Database.
