# Supplementary material for: Extracellular Vesicles of Human Periodontal Ligament Stem Cells Contain MicroRNAs Associated to Proto-Oncogenes: Implications in Cytokinesis
Source: Front Genet. 2020 Jun 4;11:582. doi: 10.3389/fgene.2020.00582 (PMC7287171; doi:10.3389/fgene.2020.00582)
Supplement: Supplementary file 1 [file Table_1.DOCX]

**Table S1.** Non-coding RNAs found in hPDLSC-derived EVs.

| **Non-coding RNAs** |
| --- |
| A1BG-AS1  AC000032.2  AC002116.8  AC002398.11  AC002398.12  AC002511.1  AC003958.2  AC004019.13  AC004069.2  AC004538.3  AC004951.6  AC005003.1  AC005042.4  AC005154.6  AC005324.6  AC005481.5  AC005523.2  AC005562.1  AC005594.3  AC005614.5  AC005618.6  AC007182.6  AC007362.3  AC007952.5  AC007952.6  AC008063.2  AC009120.6  AC009133.14  AC009133.17  AC009227.2  AC009365.3  AC009403.2  AC010127.3  AC010336.1  AC010536.1  AC010642.1  AC011484.1  AC012314.8  AC013394.2  AC016629.8  AC016747.3  AC017104.2  AC017116.11  AC018705.5  AC021016.6  AC023490.2  AC025165.8  AC027601.1  AC051649.12  AC064852.4  AC067945.4  AC068580.6  AC073842.19  AC074391.1  AC079341.1  AC079354.5  AC079586.1  AC079602.1  AC083884.8  AC084125.4  AC084219.4  AC092295.7  AC092675.3  AC093627.10  AC096574.4  AC096670.3  AC097662.2  AC099668.5  AC104024.2  AC104532.3  AC104667.3  AC104695.3  AC104809.2  AC105206.1  AC106876.2  AC109828.1  AC110619.1  AC110619.2  AC110781.3  AC114730.7  AC129492.6  AC133785.1  AC135048.13  AC137932.1  AC138035.1  AC138035.2  AC139099.5  AC144568.2  ACTA2-AS1  AD000090.2  ADIPOQ-AS1  ADIRF-AS1  AE000661.50  AF001548.5  AF001548.6  AF131215.2  AF131216.6  AF186192.1  AF230666.2  AFAP1-AS1  AGAP11  AGAP2-AS1  AJ239322.1  AJ271736.10  AL022344.5  AL031666.2  AL033381.1  AL078585.1  AL359878.1  AL360004.1  AL391421.1  AL445199.1  AL450307.1  AL590708.2  AL592494.5  ANKRD20A5P  AP000266.7  AP000320.7  AP000525.9  AP000654.4  AP001062.7  AP001372.2  AP001469.9  AP001626.2  AP003068.23  AP006222.2  AP4B1-AS1  ARAP1-AS2  ASB16-AS1  ASMTL-AS1  ATF6B  ATP1A1OS  BACH1-IT1  BAIAP2-AS1  BCRP3  BZRAP1-AS1  C10orf40  C11orf72  C12orf79  C13orf45  C14orf182  C14orf23  C14orf64  C17orf51  C17orf77  C19orf82  C19orf83  C1orf145  C1orf147  C1orf220  C21orf128  C21orf37  C21orf49  C3orf27  C3orf65  C5orf56  C5orf60  C6orf123  C6orf3  C6orf48  C7orf71  C8orf17  C8orf49  C8orf56  C9orf106  C9orf141  C9orf163  C9orf37  CACNA1C-AS1  CACTIN-AS1  CBR3-AS1  CCDC183-AS1  CCNT2-AS1  CD27-AS1  CFLAR-AS1  CIRBP-AS1  CKMT2-AS1  COL4A2-AS2  COL5A1-AS1  COLCA1  CPB2-AS1  CSAG2  CSAG3  CTA-134P22.2  CTA-384D8.36  CTB-176F20.3  CTB-189B5.3  CTB-22K21.2  CTB-25B13.12  CTB-46B19.2  CTB-51J22.1  CTB-55O6.12  CTB-59C6.3  CTB-89H12.4  CTBP1-AS2  CTC-137K3.1  CTC-228N24.3  CTC-251D13.1  CTC-265F19.1  CTC-277H1.7  CTC-301O7.4  CTC-329D1.2  CTC-338M12.6  CTC-338M12.7  CTC-345K18.2  CTC-367F4.1  CTC-425O23.2  CTC-453G23.5  CTC-459F4.3  CTC-471J1.8  CTC-479C5.10  CTC-499J9.1  CTC-512J12.4  CTC-513N18.7  CTC-518B2.9  CTC-524C5.2  CTC-548K16.5  CTC-550B14.6  CTC-558O2.1  CTD-2008P7.9  CTD-2020K17.3  CTD-2031P19.5  CTD-2047H16.4  CTD-2135D7.2  CTD-2139B15.2  CTD-2154B17.4  CTD-2154I11.2  CTD-2193P3.2  CTD-2196E14.9  CTD-2201G3.1  CTD-2210P24.4  CTD-2228K2.5  CTD-2231E14.8  CTD-2240E14.4  CTD-2251F13.1  CTD-2267D19.3  CTD-2291D10.1  CTD-2292P10.4  CTD-2303H24.2  CTD-2313N18.5  CTD-2323K18.1  CTD-2349B8.1  CTD-2368P22.1  CTD-2369P2.8  CTD-2382E5.1  CTD-2410N18.4  CTD-2517M22.14  CTD-2527I21.4  CTD-2540B15.13  CTD-2540B15.7  CTD-2540F13.2  CTD-2540L5.5  CTD-2541J13.2  CTD-2541M15.1  CTD-2547L24.3  CTD-2555A7.2  CTD-2555O16.2  CTD-2555O16.4  CTD-2574D22.2  CTD-2600O9.1  CTD-2616J11.2  CTD-2619J13.14  CTD-2619J13.8  CTD-2619J13.9  CTD-2620I22.1  CTD-2620I22.3  CTD-2630F21.1  CTD-2659N19.2  CTD-3051D23.1  CTD-3099C6.9  CTD-3105H18.14  CTD-3110H11.1  CTD-3149D2.3  CTD-3185P2.1  CTD-3193K9.4  CTD-3193O13.11  CTD-3203P2.2  CTD-3222D19.7  DGCR9  DGUOK-AS1  DHRS4-AS1  DIO3OS  DLEU1  DLEU2  DLGAP1-AS1  DNAH10OS  DNMBP-AS1  DSCR4  DTX2P1-UPK3BP1-PMS2P11  DYX1C1-CCPG1  EGFR-AS1  ENTPD1-AS1  ENTPD3-AS1  EPB42  ERVH48-1  ERVK13-1  FAM106A  FAM106CP  FAM138A  FAM138B  FAM138C  FAM138E  FAM181A-AS1  FAM182B  FAM211A-AS1  FAM215A  FAM225A  FAM225B  FAM230B  FAM74A4  FAM74A5  FAM85B  FAR2P1  FBXL19-AS1  FENDRR  FEZF1-AS1  FGD5-AS1  FKSG62  FLJ27365  FLJ45079  FOXN3-AS1  GAS5  GHRLOS  GNAS-AS1  GS1-124K5.2  GS1-251I9.4  GUSBP11  H19  HAND2-AS1  HCG15  HCG18  HCG27  HNRNPA1L2  HOXA10-AS  HOXA11-AS  HPN-AS1  hsa-mir-335  hsa-mir-490  HSD17B12  IBA57-AS1  IQCF5-AS1  IQCH-AS1  JAZF1-AS1  JPX  KANSL1-AS1  KB-1183D5.14  KB-1460A1.5  KB-1572G7.2  KB-1572G7.3  KB-1592A4.14  KCTD21-AS1  KIAA0087  KIAA1841  KIF9-AS1  LA16c-306E5.3  LA16c-313D11.12  LA16c-366D3.1  LA16c-381G6.1  LATS2-AS1  LGALS8-AS1  LINC00092  LINC00115  LINC00174  LINC00240  LINC00243  LINC00265  LINC00266-1  LINC00266-4P  LINC00273  LINC00305  LINC00452  LINC00471  LINC00479  LINC00485  LINC00544  LINC00607  LINC00632  LINC00637  LINC00654  LINC00656  LINC00657  LINC00667  LINC00677  LINC00843  LINC00851  LINC00862  LINC00894  LINC00906  LINC00910  LINC00957  LINC00958  LINC00963  LINC00969  LINC00987  LINC01001  LINC01044  LINC01090  LINC01118  LINC01119  LINC01123  LINC01128  LINC01133  LINC01140  LINC01160  LIPE-AS1  LL0XNC01-237H1.3  LL22NC03-75H12.2  LOH12CR2  LRCH4  LZTR1  MAGI2-AS3  MALAT1  MAP3K14-AS1  MATR3  MBNL1-AS1  MED14-AS1  MEG3  MIR142  MIR22HG  MIR24-2  MIR296  MIR4435-1HG  MIR497HG  MIR7-3HG  MORC2-AS1  MRGPRG-AS1  MYCBP2-AS1  NALCN-AS1  NAPA-AS1  NCAM1-AS1  NEAT1  NOP14-AS1  NPHP3-AS1  NUCB1-AS1  OIP5-AS1  OR2A1-AS1  PAXIP1-AS2  PCBP1-AS1  PDXDC2P  PI4K2B  PIGQ  PITRM1-AS1  PLCE1-AS1  POLR2J4  POTEH-AS1  PP13004  PRICKLE2-AS3  PRKAR2A-AS1  PRNT  PSMG3-AS1  PTOV1-AS1  PTPRG-AS1  RAMP2-AS1  RASAL2-AS1  RBAKDN  RBMS3-AS2  RMRP  RNF219-AS1  RP11-1006G14.4  RP11-1007G5.2  RP11-1007I13.4  RP11-1007O24.3  RP11-1020A11.2  RP11-102L12.2  RP11-106M3.3  RP11-1072C15.4  RP11-1079K10.4  RP11-1090M7.1  RP11-109P14.9  RP11-10K16.1  RP11-1100L3.7  RP11-1105G2.3  RP11-110G21.1  RP11-1112J20.2  RP11-1113L8.1  RP11-111E14.1  RP11-113D6.6  RP11-114H20.1  RP11-114H24.7  RP11-1151B14.4  RP11-1186N24.5  RP11-1191J2.2  RP11-120M18.2  RP11-124N14.3  RP11-1260E13.4  RP11-1275H24.1  RP11-128P10.1  RP11-129B22.1  RP11-132A1.4  RP11-133K1.6  RP11-1348G14.5  RP11-137L10.6  RP11-138I1.4  RP11-138P22.1  RP11-1391J7.1  RP11-144F15.1  RP11-144G6.12  RP11-144G7.2  RP11-146D12.2  RP11-148L24.1  RP11-14N7.2  RP11-14P20.1  RP11-150O12.3  RP11-150O12.6  RP11-152P17.2  RP11-156P1.3  RP11-15G16.1  RP11-161H23.5  RP11-161H23.9  RP11-161M6.2  RP11-171I2.2  RP11-173B14.5  RP11-174G6.5  RP11-182J1.12  RP11-182J1.13  RP11-188C12.3  RP11-18I14.10  RP11-192H23.7  RP11-196O2.1  RP11-197N18.2  RP11-1C1.5  RP11-203M5.8  RP11-206L10.3  RP11-206L10.9  RP11-20B24.4  RP11-20I23.8  RP11-211N8.2  RP11-212P7.2  RP11-212P7.3  RP11-214C8.2  RP11-216B9.6  RP11-216L13.18  RP11-216P16.2  RP11-219A15.4  RP11-219B4.5  RP11-219G17.4  RP11-21J18.1  RP11-223P11.2  RP11-223P11.3  RP11-227G15.3  RP11-228B15.4  RP11-22P6.3  RP11-23E10.3  RP11-23J9.4  RP11-23P13.6  RP11-247C2.2  RP11-248G5.8  RP11-253M7.1  RP11-253M7.4  RP1-125I3.2  RP11-263K19.6  RP11-264B14.2  RP11-264B17.3  RP11-264M12.2  RP11-266K22.2  RP11-266K4.9  RP11-266L9.2  RP11-269F21.3  RP11-273G15.2  RP11-274B21.1  RP11-277L2.3  RP11-280K24.4  RP11-283G6.5  RP11-283I3.6  RP11-28G8.1  RP11-290F20.3  RP11-290F24.3  RP11-291L22.4  RP11-292E2.2  RP11-296L22.8  RP11-298I3.4  RP11-298J20.4  RP11-29G8.3  RP11-2C24.4  RP11-304F15.3  RP11-304M2.6  RP11-309H21.3  RP11-312O7.2  RP11-316O14.1  RP11-317J10.2  RP11-323N12.5  RP11-324D17.2  RP11-324E6.6  RP11-333E1.1  RP11-334C17.5  RP11-334E6.3  RP11-338C15.5  RP11-33B1.4  RP11-33N16.3  RP11-341G23.4  RP11-342M1.3  RP11-343H19.2  RP11-344B2.2  RP11-345M22.3  RP11-348B17.1  RP11-34P13.13  RP11-34P13.7  RP11-351D16.3  RP11-354M1.2  RP11-356C4.3  RP11-357C3.3  RP11-35G9.3  RP11-362K14.7  RP11-362K2.2  RP11-363G10.2  RP11-365N19.2  RP11-366L20.2  RP11-367J11.3  RP11-373L24.1  RP11-375N15.2  RP11-378A13.1  RP11-378J18.8  RP11-37C7.1  RP11-381K20.2  RP11-382J12.1  RP11-383C5.3  RP11-383H13.1  RP11-383M4.2  RP11-383M4.6  RP11-384F7.2  RP11-386G11.10  RP11-386G11.5  RP11-386G21.2  RP11-387D10.2  RP11-388M20.1  RP11-388M20.6  RP11-38L15.2  RP11-38L15.8  RP11-392B6.1  RP11-394J1.2  RP11-399D6.2  RP11-3P17.5  RP11-400F19.6  RP11-400F19.8  RP11-406H23.2  RP11-414J4.2  RP11-415C15.2  RP11-416N2.4  RP11-417J8.6  RP11-419C23.1  RP11-420L9.5  RP11-423P10.2  RP11-425A6.5  RP11-426A6.5  RP11-429E11.3  RP11-434D2.3  RP11-434D2.7  RP11-435B5.5  RP11-438B23.2  RP11-439A17.7  RP11-440L14.4  RP11-443C10.1  RP11-448A19.1  RP11-449L23.2  RP11-449P15.1  RP11-449P15.2  RP11-44N11.1  RP11-452K12.7  RP11-452L6.5  RP11-458D21.6  RP11-45M22.3  RP11-462G12.1  RP11-464F9.1  RP11-464F9.21  RP11-467L20.10  RP11-468E2.9  RP11-46A10.5  RP11-46J23.1  RP11-474G23.3  RP11-475I24.3  RP11-478C19.2  RP11-47I22.3  RP11-481G8.2  RP11-493P1.2  RP11-496I9.1  RP11-497E19.2  RP11-505E24.2  RP11-50E11.3  RP11-510C10.3  RP11-510H23.1  RP11-510N19.5  RP11-513M16.7  RP11-513N24.1  RP11-517A5.4  RP11-517H2.6  RP11-519G16.3  RP11-547D24.1  RP11-548H3.1  RP11-552I14.1  RP11-553A10.1  RP11-555J4.4  RP11-561E1.1  RP11-566K11.4  RP11-573D15.9  RP11-574K11.28  RP11-582J16.5  RP11-583F2.2  RP11-586D19.2  RP11-589P10.7  RP11-592B15.3  RP11-592B15.9  RP11-597A11.6  RP11-598F7.1  RP11-598F7.5  RP11-603J24.5  RP11-605F22.1  RP11-618G20.1  RP11-61I13.3  RP11-625H11.2  RP11-640M9.1  RP11-640N11.2  RP11-641A6.2  RP11-649A18.12  RP11-649E7.5  RP11-650L12.2  RP11-659E9.2  RP11-65J3.1  RP11-65L3.2  RP11-661A12.7  RP11-661A12.9  RP11-66B24.4  RP11-66D17.3  RP11-66N24.3  RP11-66N24.4  RP11-672L10.2  RP11-67A1.2  RP11-67L3.5  RP11-686D22.8  RP11-69E11.8  RP11-6O2.4  RP11-706O15.1  RP11-706O15.3  RP11-706O15.5  RP11-706O15.7  RP1-170O19.17  RP11-712L6.5  RP11-713P17.3  RP11-713P17.4  RP11-717H13.1  RP1-171K16.5  RP11-723O4.2  RP11-727A23.7  RP11-731C17.2  RP11-73E17.2  RP11-744N12.3  RP11-750H9.5  RP11-752D24.2  RP11-752G15.3  RP11-757G1.6  RP11-758N13.1  RP11-75C10.7  RP11-76C10.5  RP11-770J1.4  RP11-774O3.3  RP11-778O17.4  RP11-77H9.6  RP11-77K12.8  RP11-781P6.1  RP11-782C8.2  RP11-782C8.5  RP11-783K16.10  RP1-178F15.4  RP1-178F15.5  RP11-796E2.4  RP11-798G7.6  RP11-799B12.4  RP11-802E16.3  RP11-812E19.3  RP11-81K13.1  RP11-822E23.6  RP11-822E23.8  RP11-829H16.3  RP11-830F9.6  RP11-834C11.3  RP11-847H18.2  RP11-849H4.4  RP11-84C10.4  RP11-856M7.2  RP11-85L21.4  RP11-85O21.2  RP11-863K10.7  RP11-86H7.6  RP11-875O11.1  RP11-903H12.3  RP11-90K6.1  RP11-927P21.1  RP11-930P14.1  RP11-932O9.8  RP11-936I5.1  RP11-95K23.3  RP11-96C23.5  RP11-977G19.14  RP11-983P16.4  RP11-989E6.3  RP11-99J16__A.2  RP1-228P16.4  RP1-257A7.4  RP1-266L20.9  RP1-283E3.8  RP1-30M3.5  RP13-1039J1.4  RP13-212L9.1  RP13-329D4.3  RP13-452N2.1  RP13-608F4.8  RP1-37C10.3  RP1-37N7.1  RP1-37N7.3  RP13-890H12.2  RP1-43E13.2  RP1-59M18.2  RP1-69M21.2  RP3-323P13.2  RP3-330M21.5  RP3-337O18.9  RP3-395M20.8  RP3-449M8.9  RP3-467K16.2  RP3-467L1.4  RP3-508I15.9  RP4-545C24.1  RP4-548D19.3  RP4-569M23.2  RP4-598G3.1  RP4-605O3.4  RP4-622L5.2  RP4-639F20.1  RP4-647C14.2  RP4-669L17.2  RP4-680D5.2  RP4-728D4.2  RP4-734G22.3  RP4-735C1.4  RP4-751H13.7  RP4-756G23.5  RP4-758J18.13  RP4-758J18.2  RP4-773N10.4  RP4-778K6.3  RP4-784A16.2  RP4-785G19.5  RP4-798P15.3  RP4-800J21.3  RP5-1021I20.5  RP5-1024G6.2  RP5-1024N4.4  RP5-1039K5.17  RP5-1073O3.7  RP5-1112D6.8  RP5-1139B12.2  RP5-1139B12.3  RP5-1142A6.2  RP5-1142A6.7  RP5-1142A6.9  RP5-1165K10.2  RP5-1184F4.5  RP5-1198O20.4  RP5-828H9.1  RP5-837M10.4  RP5-857K21.2  RP5-864K19.4  RP5-902P8.12  RP5-906A24.2  RP5-940J5.6  RP5-940J5.9  RP5-947P14.1  RP5-981O7.2  RP6-206I17.1  RP6-206I17.2  RP6-42F4.1  RPA3-AS1  RPL34-AS1  RPPH1  RPS17L  RRN3P2  RUSC1-AS1  SBF2-AS1  SCARNA10  SCARNA2  SERTAD4-AS1  SH3BP5-AS1  SLC16A6P1  SLC2A1-AS1  SLX1A-SULT1A3  SLX1B-SULT1A4  SMCR5  SNHG1  SNHG14  SNHG15  SNHG16  SNHG3  SNHG7  SNORA40  SNORA67  SNORD109A  SNORD116-20  SNORD3D  SOCS2-AS1  SOD2  SPACA6P-AS  SRD5A3-AS1  SRP14-AS1  ST7-OT4  STAG3L5P-PVRIG2P-PILRB  STARD7-AS1  STEAP3-AS1  SULT1A4  TAPSAR1  TBC1D3P1-DHX40P1  TCL6  TERC  TFAP2A-AS1  THAP7-AS1  THBS1  TLX1NB  TMEM78  TMEM99  TNRC6C-AS1  TPT1-AS1  TPTEP1  TRAF3IP2-AS1  TRHDE-AS1  TSIX  TTC28-AS1  TTLL10-AS1  TTN-AS1  U91328.19  UBA6-AS1  UPK1A-AS1  USP2-AS1  VARS2  VIM-AS1  VPS9D1-AS1  WDR11-AS1  WT1-AS  XIST  XXbac-B33L19.3  XXbac-B476C20.13  XXcos-LUCA11.4  Z83844.1  ZEB2  ZEB2-AS1  ZNF252P-AS1  ZNF280D  ZNF337-AS1  ZNF503-AS2  ZNF667-AS1  ZNF833P  ZSCAN16-AS1  ZSWIM8-AS1 |
